# Use of a Creative Problem Solving (CPS) Approach in a Senior Thesis Course to Advance Undergraduate Publications

**DOI:** 10.3389/fpsyg.2019.00749

**Published:** 2019-04-09

**Authors:** Mareike B. Wieth, Andrea P. Francis, Andrew N. Christopher

**Affiliations:** Department of Psychological Science, Albion College, Albion, MI, United States

**Keywords:** undergraduate research, mentoring undergraduate students, faculty collaborations, student development, graduate school preparation

We outline a creativity-based course model for supervising and promoting undergraduate research at a small liberal arts college of about 1,600 undergraduate students, with no graduate offerings. This approach could easily be modified and implemented at weekly brownbag or joint laboratory meetings at similar and larger types of schools. At our institution this course is required of all psychology research thesis students (on average 8 per year) and requires the cooperation of the students, their thesis supervisors, and the course instructor. In part because of this course, during the past 20 years, our department faculty have published a total of 47 publications with undergraduates in peer-reviewed outlets such as *Personality and Individual Differences, Psychology of Music, Psychology of Women Quarterly*, and *Sex Roles*. Importantly, according to PsycINFO, these undergraduate-generated publications have garnered more than 500 citations, attesting to the impact that undergraduate research can have on the larger field in terms of knowledge generation. In addition to impactful peer-reviewed publications, our undergraduate students have presented 163 posters at national conferences such as the Association for Psychological Science, Society for Personality and Social Psychology, Society for Neuroscience, and Psychonomic Society. Below we outline how our senior thesis course stimulates the creative dissemination of knowledge that is required during the publication process.

Our senior thesis course structure is based on the Creative Problem Solving (CPS) framework, a well-known and validated approach to creativity enhancement in educational settings. This approach emphasizes creative and critical thinking in instruction—both at an individual and a group level (Baer, 1988; Isaksen et al., 1994; Treffinger et al., 2006). In the CPS framework, creative thinking occurs when a problem or challenge is considered from many different perspectives, which leads to a multitude of possible solutions or answers (this is also known as divergent thinking—see Wieth and Francis, 2018 for a review). In this stage of creative problem solving, many original solutions or answers are desired (Boynton, 2001). The second aspect of the CPS framework is critical thinking (also known as convergent thinking—see Wieth and Francis, 2018 for a review). After generating possible solutions to a problem or challenge, it is essential for the student to converge on a single most useful solution for that particular problem or challenge (Campbell, 1960; Mednick, 1962; Lundsteen, 1986; Amabile, 1988; Mumford, 2003; Sternberg, 2010). In this article, we outline how using the CPS framework in our senior thesis research course has prepare and enable our students to thrive during the publication process.

Reiterative critical feedback of written and oral production is an essential component of the CPS approach used in our senior thesis course. Written assignments in this course are no different than what advisors usually ask of their thesis students (e.g., complete a draft of the Introduction or Method), but in keeping with the CPS approach, each written component goes through a cycle of creative and critical feedback from several peers during class. As can be seen from the most recent syllabus, available as Supplementary Material, students must bring their writing to class four times across the semester to be reviewed by peers. Collaboration, social support (John-Steiner, 2000), and honest critique (Nemeth et al., 2001) are viewed as key factors in creative breakthroughs. Therefore, each time peer-review occurs, students are asked to provide and receive constructive feedback from at least two peers in the course. The instructor of the course orchestrates the pairings to ensure that students receive a diverse set of feedback. Typically, a student is paired with a classmate using a similar research approach AND with a classmate using a very different research approach. At a liberal arts college, there is often only one faculty member per psychological discipline (e.g., cognitive psychology), so a student may be working with an advisor that is a cognitive psychologist but receiving feedback from a student working with a social psychologist or neuroscientist. Receiving feedback from someone in a different area of psychology often encourages more divergent thinking and helps students understand the greater context of their research. In other words, the first step in the peer-review process is designed to encourage more creative thinking.

The second part of the CPS framework employed during peer-review in the senior thesis course is designed to encourage critical thinking by having students to practice converging on a best solution to a problem or challenge. For example, during the peer-review process, each student must decide which suggestions are appropriate and helpful for their project and which suggestions are counter to the purpose of the project. However, unlike when students receive feedback from their faculty advisor or an outside faculty member, students feel more comfortable critically evaluating the suggestions from their peers. This provides an excellent mechanism for students to practice critical evaluation after being exposed to a wide range of feedback.

Another way we encourage critical thinking in our course is to scaffold students' research by having students make four platform presentations, each with a different focus for a different audience. During the class, students make two 20-min platform presentations to their peers and other faculty. After receiving feedback from peers on the written portion of the Introduction and Method, the student must give a presentation that covers the Introduction and Method sections. After receiving feedback from peers on the written portion of the Results and Discussion, the student must give a presentation that covers the Results and Discussion sections. After presenting for the allotted time, there is approximately 15 min of discussion devoted to each student's project and presentation. The student's research advisor along with other faculty in the department attend these presentations throughout the semester and provide feedback in an intellectually safe environment. The attendance of faculty other than the instructor is of critical importance during these presentations and serves several purposes. In addition to instruction and practice of psychology presentation skills, the discussion after the presentation allows the faculty to model appropriate conflict resolution and problem solving strategies. Research has shown that fostering an environment where honest and thoughtful dissent is accepted and appreciated enhances productivity and fosters creativity (Nemeth et al., 2004). At first students are often surprised and perhaps a bit intimidated when they experience two or more faculty members debating some aspect of their project, but by the end of the semester, students are more comfortable joining in the debate in a meaningful and appropriate way. Modeling critical and thoughtful responses not only leads our students to hone their thinking and presentation skills, it also provides them essential experience for responding to comments during the peer-review publication process.

As a culminating experience, students must also present their work in two other venues: a regional undergraduate psychology conference and a college-wide research symposium. The purpose of these myriad presentations is for students to learn what components of all the work they have done are essential for presentation to different audiences. In other words, students must converge on a best solution depending on the audience to whom they are presenting. In each situation, the student must modify their presentation for the audience. For many of our research students, this is their first foray into professional psychology meetings, so rather than going initially to a national meeting, we require students in the course to present at regional undergraduate psychology research conference held each spring. This meeting provides students with the opportunity to receive additional reviews of their work, this time from psychology faculty and other psychology majors at different schools who may bring perspectives different from those in our department. To develop more critical feedback response skills, students are required to present at a college-wide symposium given to faculty and students outside the psychology department. For the all-college symposium, students learn how to present their research in a very different way than they have done for their theses and presentations to “psychology-oriented” audiences. For instance, although the importance of basic research into personality may be self-evident to psychologists, it is less obvious to faculty and students not trained in our discipline. Thus, students need to, again, think about their work from a wider perspective, this time including a very diverse audience, to find the most effective way of presenting their research. Much like the peer-review process often provides researchers with different and sometimes even conflicting suggestions; these presentations help students see their own work from multiple perspectives and forces them to choose a presentation and feedback response format that best fits the audience.

Our course outlined here prepares students for what is required during publication by exposing our students to diverse feedback from students, psychology faculty, and college-wide faculty. This is similar to the sundry reviews authors often receive after submitting a manuscript. In addition, our creativity focused classroom model also promotes critical thinking, a fundamental component of creativity, as outlined by the CPS framework (Isaksen et al., 1994). By teaching students to evaluate feedback from a variety of individuals and adjust their presentations to various audiences, we are encouraging critical thinking that helps students understand the importance of finding the best way to present their research. Furthermore, these critical thinking skills help students not get overwhelmed by reviews of their manuscript and the, often many, demands reviewers make. Providing this course to all thesis students in our department has enabled us to teach students more about the research and publication process, allowed us to include more students on publications and national presentations that arise from their own research, and support our fellow faculty in their senior thesis advising endeavors by ensuring that their students meet their goals and deadlines. Using the CPS framework in a course, does take a certain amount of effort and collaboration from advisors, students, and other faculty, but we, and our fellow faculty in the department, believe that those efforts are well-spent as our senior thesis students' work often turns into influential publications in their respective fields.

## Author Contributions

All authors listed have made a substantial, direct and intellectual contribution to the work, and approved it for publication.

### Conflict of Interest Statement

The authors declare that the research was conducted in the absence of any commercial or financial relationships that could be construed as a potential conflict of interest.
