# Supplementary material for: Use of a Creative Problem Solving (CPS) Approach in a Senior Thesis Course to Advance Undergraduate Publications
Source: Front Psychol. 2019 Apr 9;10:749. doi: 10.3389/fpsyg.2019.00749 (PMC6465638; doi:10.3389/fpsyg.2019.00749)
Supplement: Supplementary file 1 [file Table_1.DOCX]

Syllabus for Psychology 416, Spring 2018

Senior Research Seminar

**Meeting Time:** Wednesday 4:15 – 7:15pm, Olin 341

# Professor: Mareike Wieth (Mar-i-ka Veet)

**Office:** 325C Olin

**Email:** [mwieth@albion.edu](mailto:wiethmar@msu.edu)

**Office Hours:** Tuesday 10:30 - 11:30am, Thursday 3 – 4:30 pm, or by appointment

**Prerequisite**

Senior standing with intention to complete a major research project or departmental/ college honors thesis.

**Course Description**

This course guides students completing a senior thesis through all aspects of the research process. It will focus on data analysis, interpretation and reporting on the results of student research projects. We will consider both theoretical and practical research issues.

**Honor and Integrity**

As an academic community, Albion College is firmly committed to honor and integrity in the pursuit of knowledge. Therefore, as a member of this academic community, each student acknowledges responsibility for his or her actions and commits to the highest standards of integrity. In doing so, each student makes a covenant with the college not to engage in any form of academic dishonesty, fraud, cheating, or theft.”

**Course Requirements**

**Class Presentations**

Throughout the semester you will make **3 presentations in class**. **Each presentation is worth 100 points.**

- The first presentation will introduce us to your theoretical area and methodology. It should be about 20 minutes and include information that will appear in the introduction and method sections of your thesis. You should also be prepared to answer questions for an additional 15 minutes.
- The second presentation will be another 20 minute presentation. This presentation will be of your data analyses and interpretation/discussion of these results. It will include information that will appear in the results and discussion sections of your thesis. Again you should be prepared to answer questions for an additional 15 minutes.
- The third presentation will be a 12 minute practice conference presentation. (Timing will be strictly enforced for this presentation.)

**Conference Attendance and Presentation**

- You must **give a presentation at the Elkin Isaac Research Symposium and an appropriate research conference.** An example of an appropriate local research conference is the Michigan Undergraduate Psychology Research Conference on April 14 at Ferris State University. Other local or national conferences can also fulfill this course requirement; the conference however must be approved by your adviser. Fulfillment of this two-part requirement counts for **100 points** toward the course point total.

**Proofreading Swaps**

There will be 3 times throughout the semester where you will be required to bring sections of your research/thesis paper to class. This assignment requires that you have a substantial amount of writing completed on the sections that will be reviewed that day. You will be asked to bring 3 printed out copies of your writing to class. You will then swap your writing with other students in the class and provide comments on their writing. Fulfillment of this requirement contributes **150 points toward** the course point total. Your points will be based on the appropriateness of your written work and the appropriateness of the comments you provide to the other students.

**Assignments**

Throughout the semester you will be asked to complete several small assignments designed to help you complete your thesis. You will generally be given 1 week to complete these assignments. The assignments will contribute **50 points** toward your course grade.

**Attendance and Participation**

Because this is a seminar, your attendance and participation is even more essential than it is in a “normal” class. Participation in discussions allows you to provide essential feedback to your classmates. Because you are a naïve listener (relative to the student presenter and his or her advisor), you will have perspectives that other people lack. We need to hear your comments and suggestions. There are **200 points** available for attendance and participation.

**Please NOTE**

Behaviors that will lead to an automatic loss of 50 points each time they occur:

- Not being ready for your presentation on the day assigned.
  - Unless this has been cleared with me at least 4 days before the presentation date.
  - You may switch dates with another student. You are responsible for finding a student that is willing to switch with you and you MUST notify me of the change at least 4 days before the presentation date.
- Skipping class (without an appropriate excuse such as a job/grad school interview)
  - If you need to miss class please notify me at least 7 days before the date.
- Playing on your laptop, reading, or engaging in any other behavior not related to the purpose of the class especially during other student’s presentations.
  - Your classmates are counting on your feedback; please help them as they will help you.
- Spacing out and/or daydreaming during other student’s presentations.
- Again, your classmates are counting on your feedback; please be a participant not just a body.

**Accommodations:**

- If you have a disability and may require accommodations or modifications in class instruction or course-related activities, please contact the Learning Support Center (LSC) staff who can arrange for reasonable accommodations for students who provide documentation of their disability/condition. If you are presently registered with the LSC and have requested accommodations through the LSC for this semester, please plan to meet with me as early as possible to discuss the best way to implement these accommodations in this class. The LSC is located on the third floor of the Seeley Mudd library or call 517-629-0825.

### Grading:

Your presentations, proofreading swaps, assignments, conference attendance and presentation, and attendance and participation points will be combined into a final score out of a possible 800, and this final score will be converted into your final grade. Your total will be rounded to the nearest whole number.

**For example, if your presentation grades were 85/100, 85/100, 90/100, your proofreading swap grade was a 125/150, you got a 90/100 for your conference attendance and presentation, a 46/50 on your assignment, and a 180/200 for your attendance and participation, your final score would be: (85+85+90+125+90+46+180)/800 = 701/800 = 87.6 (A final score of 87.4 or lower is rounded down to 87, a score of 87.5 or higher would be rounded up to 88)**

The following scale will be used to convert your final score into your final grade. For example, 88 would be a 3.3. Once you have received all of your grades you should be able to add up all your scores to compute your final grade.

| **Number of Points** | **Grade** |
| --- | --- |
| 93 and higher | 4.0 (A) |
| 90 - 92 | 3.7 (A-) |
| 87 - 89 | 3.3 (B+) |
| 83 - 86 | 3.0 (B) |
| 80 - 82 | 2.7 (B-) |
| 77- 79 | 2.3 (C+) |
| 73 - 76 | 2.0 (C) |
| 70 – 72 | 1.7 (C-) |
| 65 - 69 | 1.3 (D+) |
| 56 - 64 | 1.0 (D) |
| 55 or less | 0.0 (F) |

**TENTATIVE Class Schedule**

NOTE: **this schedule is subject to change depending on the needs of the class.**

Jan. 17 Introduction – discuss course, presentations, and thesis process

Jan. 24 Proofreading Swap – Method (or Background Information)

Jan. 31 Presentation #1 – **at least three students**

****Friday, Feb. 2 - Elkin Isaac nominations due***

Feb. 7 Presentation #1 – **at least three students**

****Friday, Feb. 9 - Elkin Isaac Abstracts due***

Feb. 14 Proofreading Swap – Introduction & Method

Feb. 21 Presentation #2 – **at least three students**

Feb. 28 Presentation #2 – **at least three students**

March 7 **No class - Spring Break**

****Friday, Mar. 9 – MUPRC submissions due****

March 14 Proofreading Swap – Results & Discussion

March 21 Proofreading Swap – All sections (including References, Graphs etc.)

March 28 Defense Week and Writing

****Monday, April 2, 4:00 p.m. - Thesis signature page due to**

**Renee Kreger in the Honors Institute! ****

April 4 Conference Presentation – **at least three students**

****Monday, April 9, 4:00 p.m. - Final thesis due to Renee Kreger in Honors! ****

April 11 Conference Presentation – **at least three students**

****Saturday, April 14: Michigan Undergraduate Psychology Research Conference****

April 18 Passing the torch

**** Thursday, April 19, Elkin Isaac Day!**

April 25 Reflections upon Thesis Process – Legacy Project

May 2 Legacy Project due
